# Implants as anchorage for the correction of localized dental malocclusion: a retrospective clinical study

**DOI:** 10.3389/fbioe.2025.1703249

**Published:** 2025-12-04

**Authors:** Ying-Wen Hu, Yin-Xin Zeng, Yu-Xuan Zhang, Peng-Yu Lai, Ran Liang, Yi-Qi Chen, Hai Huang, Guan-Sheng Guo

**Affiliations:** 1 Department of Stomatology, Affiliated Stomatological Hospital of Jinan University (Daliang Hospital), Foshan, Guangdong, China; 2 School of Stomatology, Jinan University, Guangzhou, China; 3 Clinical Research Platform for Interdiscipline of Stomatology, Jinan University, Guangzhou, China

**Keywords:** dental implants, orthodontic anchorage procedures, malocclusion, treatment outcome, tooth movement techniques

## Abstract

**Background:**

Tooth loss accompanied by adjacent tooth inclination or rotation presents a biomechanical challenge in combined orthodontic–implant rehabilitation. Whether “implant-first anchorage” provides superior clinical efficiency and biological benefits compared with the conventional “orthodontics-first then implant” pathway remains uncertain.

**Methods:**

A retrospective cohort study was conducted based on cases treated between January 2019 and December 2024 by the Departments of Implantology and Orthodontics at the Affiliated Stomatological Hospital of Jinan University. Patients with partial edentulism and localized malalignment were allocated into two cohorts according to the actual treatment pathway received: implant-first (n = 20) versus orthodontics-first (n = 20). Clinical data were extracted from medical records and radiographs. Primary outcomes included treatment duration, cost, and patient-reported satisfaction (VAS). Secondary outcomes comprised periodontal parameters (mPLI, SBI, PD), adjacent tooth inclination, alveolar crest height, and number of visits.

**Results:**

Compared with the orthodontics-first cohort, the implant-first cohort showed a markedly shorter time for local alignment (5.00 ± 1.25 vs. 11.78 ± 2.35 months, *P* < 0.001), lower overall cost (3,000.00 ± 0.00 vs. 6,100.00 ± 680.56 RMB, *P* < 0.001), fewer visits (8.20 ± 1.32 vs. 15.10 ± 2.49, *P* < 0.001), and higher satisfaction (8.05 ± 1.32 vs. 6.10 ± 1.68, *P* < 0.001). Periodontal metrics were consistently more favorable in the implant-first cohort (mPLI 1.20 ± 0.36 vs. 3.05 ± 0.50; SBI 0.96 ± 0.46 vs. 1.89 ± 0.39; PD 2.09 ± 0.37 vs. 2.67 ± 0.33; all *P* < 0.01). Changes in adjacent tooth inclination and space gain were comparable between cohorts (*P* > 0.05). Alveolar crest resorption was lower in the implant-first cohort (0.36 [0.06, 0.85] vs. 1.25 [0.54, 2.24] mm, *P* = 0.012).

**Conclusion:**

When appropriately indicated, an implant-first anchorage pathway yields superior efficiency, lower cost, and better periodontal conditions compared with the conventional orthodontics-first approach, without compromising space control or tooth movement quality.

## Highlights

- Implants serve as reliable absolute anchorage for localized malocclusion correction.
- Implant-first strategy markedly reduces treatment duration and clinical visits.
- Implant-first strategy lowers overall treatment cost while maintaining comparable tooth movement and less alveolar bone loss.

## 1 Introduction

Partial edentulism accompanied by adjacent tooth malposition (tilting, rotation, supraeruption, or displacement) is a common challenge in both prosthodontic and orthodontic practice (Lorente et al., 2021). The loss of occlusal support in edentulous areas often leads to alveolar ridge resorption and abnormal tooth positions, impairing both mastication and esthetics, while complicating prosthetic rehabilitation (Turley, 2020). Conventionally, treatment adheres to the “orthodontics-first” principle (Altieri et al., 2020), in which orthodontic appliances are applied to open adequate space for implant placement, reestablishing proper axial alignment and interproximal relationships, followed by implant restoration (Yuan et al., 2025; Tamer et al., 2020). While effective in tooth alignment, this approach has limitations: orthodontic treatment substantially prolongs the overall timeline (12–18 months on average), auxiliary devices (e.g., coil springs, mini-screws) increase costs (Naik et al., 2020; Pujol, 2021), plaque accumulation around appliances elevates periodontal risks, and frequent visits increase patient burden (Magkavali-Trikka et al., 2018).

With the integration of implantology and orthodontics, implants as anchorage have emerged as an alternative solution (Yehya et al., 2023). Their absolute anchorage, established via osseointegration, enables direct orthodontic force application, potentially reducing treatment duration, surgery frequency, and costs (Zhou et al., 2020). However, existing evidence remains scarce and fragmented, and there is a lack of controlled data directly comparing implant-first and orthodontics-first pathways under comparable clinical indications.

Against this background, the present retrospective cohort study compared the clinical efficiency, periodontal impact, biomechanical outcomes, and patient-centered endpoints of an implant-first anchorage strategy versus the conventional orthodontics-first approach in patients presenting with localized malalignment adjacent to an edentulous space. By analyzing multi-dimensional clinical indicators from real-world clinical records, this study aimed to provide evidence-based guidance for sequencing decisions in combined orthodontic-implant care.

## 2 Materials and methods

### 2.1 Study design

This retrospective cohort study reviewed consecutive patients treated between January 2019 and December 2024 at the Departments of Implantology and Orthodontics, Affiliated Stomatological Hospital of Jinan University. The protocol was approved by the institutional ethics committee (Approval No. 2022-05).

### 2.2 Inclusion and exclusion criteria

Eligible cases met all of the following criteria: age 18–80 years; single- or double-tooth edentulism for ≥3 months; localized adjacent malalignment (inclination, rotation, or vertical displacement) requiring orthodontic correction; bone quality classified as type II–III by Lekholm & Zarb; and stable periodontal condition (PD ≤ 4 mm, BOP <10%). Exclusion criteria included active periodontitis, major bone augmentation requirements, orthognathic indications, prior head-neck irradiation, uncontrolled systemic conditions affecting bone metabolism, long-term bisphosphonate use, parafunction, pregnancy/lactation, and incomplete follow-up.

### 2.3 A-priori sample size justification

Although this study was retrospective in nature, an a-priori sample size estimation was performed to ensure sufficient statistical power for the primary outcome, which was the inclination angle measured at the end of treatment. According to relevant literature (Bellini-Pereira et al., 2020), the mean inclination angle was 110.58° ± 8.54° in the implant-first group and 100.54° ± 6.53° in the orthodontics-first group. Assuming a two-sided significance level α = 0.05, power (1–β) = 0.90, and an equal group allocation ratio of 1:1, the required sample size per group was calculated using PASS 21.0.3 software according to the following formula:

$$n_{1}=n_{2}=\frac{2{\left(Z_{\alpha /2}+Z_{\beta }\right)}^{2}\times \sigma^{2}}{{\left(\mu_{2}-\mu_{1}\right)}^{2}}$$

The computed minimum sample size was 13 subjects per cohort. Considering a 20% anticipated attrition rate, the final target sample size was set at 17 patients per cohort. The actual analyzed dataset comprised 20 cases per group, exceeding the minimum requirement.

### 2.4 Cohort allocation

**FIGURE 1:**
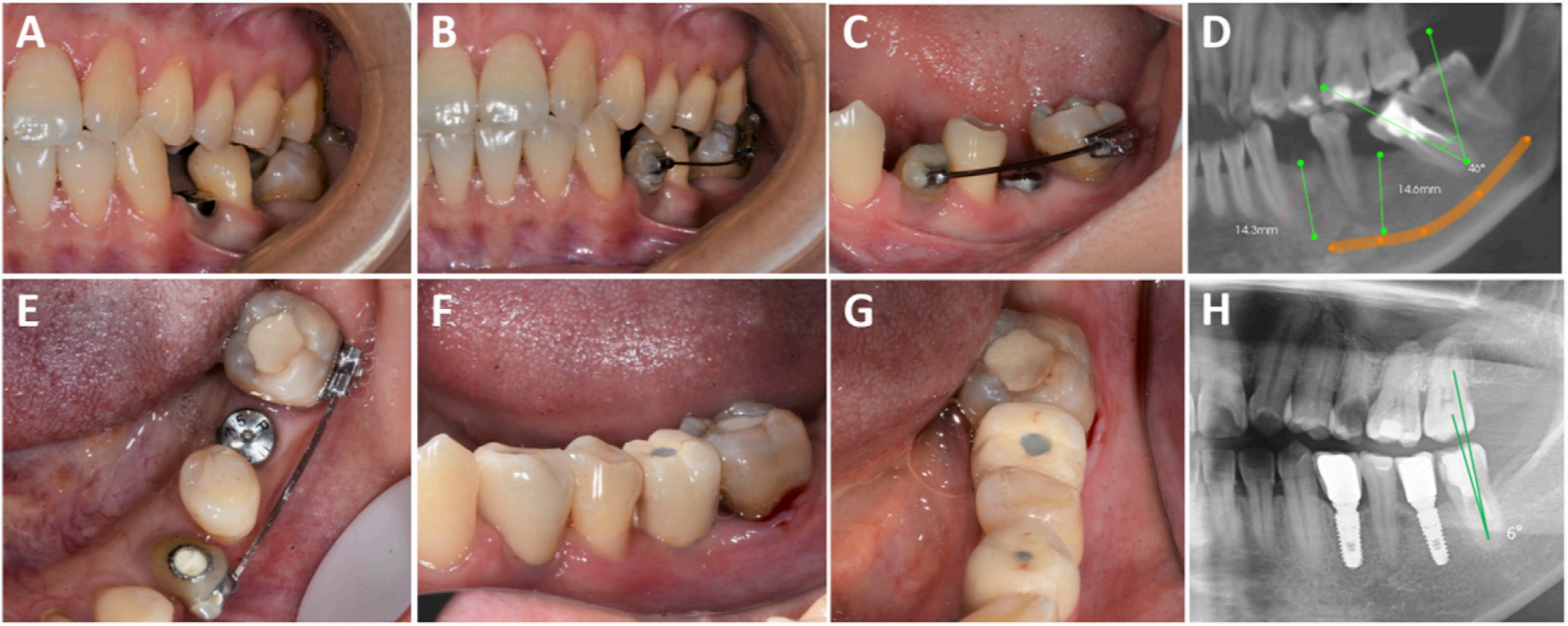
Clinical procedures of the implant-first cohort. **(A)** Implant placement in the edentulous area with localized dentition malformation; **(B,C)** Placement of a temporary abutment on tooth 34 with buccal tube bonding, followed by sequential use of 0.018 NT, 0.016 × 0.022 NT (coil spring applied to upright tooth 37 starting from this archwire), 0.019 × 0.025 NT, and 0.019 × 0.025 SS archwires; **(D)** Preoperative CBCT measurement; **(E)** Sufficient restorative space confirmed for teeth 34 and 36; **(F,G)** Prosthesis placement; **(H)** Postoperative CBCT after prosthesis placement on teeth 34 and 36.

Patients were allocated into two cohorts based on documented treatment sequencing:

Implant-first cohort (n = 20): patients underwent the following protocol. After baseline examination and preoperative documentation, including intraoral photographs and panoramic radiographs, implant surgery was performed under local infiltration anesthesia and aseptic conditions. Depending on clinical need, either flap or flapless approaches were used, and osteotomies were prepared freehand with stepwise drilling. Osstem implants (diameter and length selected based on bone availability) were placed with insertion torque ≥35 Ncm, achieving primary stability, and healing abutments were connected before suturing when applicable. Postoperatively, patients received metronidazole (0.4 g, tid for 3 days) and ibuprofen sustained-release capsules (300 mg as needed for 3 days), with suture removal at 7–10 days. Patients were instructed to avoid excessive functional loading for at least 4 weeks. After 3 months, when soft tissue healing was confirmed, temporary abutments were connected under local anesthesia and used as anchorage. Orthodontic brackets (MBT system, 0.022-inch slot) were bonded to one or two adjacent malposed teeth, and a segmental archwire was applied, with one end attached to the temporary abutment and the other to the orthodontic brackets or buccal tubes. Light continuous forces were applied for movements such as uprighting, rotation correction, or minor translation. Monthly follow-up appointments were scheduled to evaluate force levels, tooth movement progress, implant stability, soft tissue health, and oral hygiene. Archwires were sequentially upgraded (0.014 NiTi, 0.018 NiTi, 0.016 × 0.022 NiTi, 0.019 × 0.025 stainless steel) as needed, with appropriate adjustment of force direction and magnitude (Zhou et al., 2020). Once adjacent teeth achieved optimal positions, orthodontic forces were discontinued, the temporary abutments and orthodontic appliances were removed, and definitive restorations were fabricated (all-ceramic or porcelain-fused-to-metal crowns). The prostheses were delivered after occlusal adjustment to eliminate interferences, and retainers were provided (Catherine et al., 2023) (Figure 1).

**FIGURE 2:**
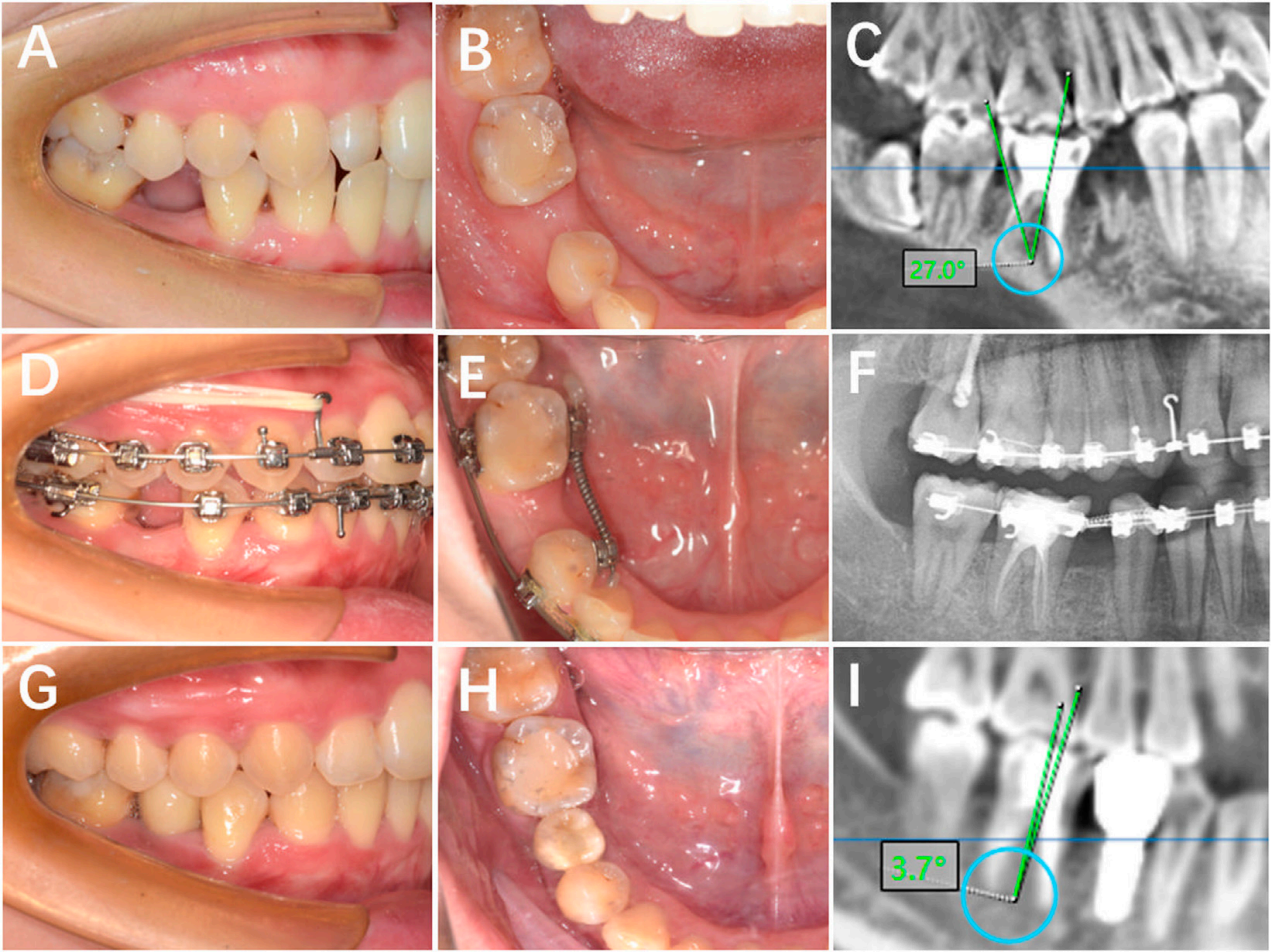
Clinical procedures of the orthodontics-first cohort. **(A,B)** Occlusal and buccal views of tooth 45 missing; **(C)** Radiographic image of tooth 45 missing; **(D,E)** Intraoral images showing orthodontic space opening for tooth 45; **(F)** Radiographic image of orthodontically opened space for tooth 45; **(G,H)** Completed crown restoration after orthodontic treatment for tooth 45; **(I)** Radiographic image after prosthesis placement on tooth 35.

Orthodontics-first cohort (n = 20): patients followed the conventional protocol. After initial documentation, fixed appliances (MBT system, 0.022-inch slot) were bonded to the malposed and adjacent teeth (Peng et al., 2024), and sequential archwires (0.014 NiTi, 0.018 NiTi, 0.016 × 0.022 NiTi, 0.019 × 0.025 stainless steel) were used for leveling and alignment. Open-coil springs and auxiliary devices were applied to the edentulous area to gradually expand the interproximal space, using neighboring teeth as anchorage (Reddy et al., 2008; Sbricoli et al., 2022), until the space exceeded the planned implant diameter (Zhao et al., 2024). Monthly reviews were scheduled to assess force application, space gain, anchorage stability, and oral hygiene. Once sufficient space was achieved and stabilized, implant placement was performed in the edentulous area with Osstem implants (insertion torque ≥35 Ncm, primary stability achieved), followed by healing abutment placement and suturing. Postoperative management mirrored that of the experimental group. A conventional healing period of 3–6 months (mandible: 3 months; maxilla: 4–6 months) was observed, during which orthodontic appliances maintained space. Following osseointegration, definitive restorations were fabricated and delivered, and retainers were provided (Catherine et al., 2023) (Figure 2).

Malocclusion classification at baseline showed that each cohort contained one patient (5%) with Angle Class II malocclusion, while the remaining 19 patients (95%) in each cohort exhibited Angle Class I malocclusion patterns. All surgical procedures were performed by the same experienced implantologist, and all orthodontic procedures were completed by the same orthodontic specialist. Cone-beam computed tomography (CBCT; model 30800026, Kunya Medical) was performed for all patients before and after treatment, and measurements were taken using OnDemand3D Dental software. Across both groups, all patients received standardized oral hygiene education throughout treatment, including the Bass toothbrushing technique and the use of dental floss or interdental brushes.

### 2.5 Outcome measures and data collection

Primary outcome measures included treatment duration, defined as the interval from initiation to the achievement of adequate space for prosthetic restoration, recorded in months, and treatment cost in Chinese yuan, which included orthodontic phase expenses. Secondary outcomes included changes in alveolar ridge height, measured as the vertical distance between the alveolar crest and fixed reference points such as the mandibular canal or sinus floor; mesiodistal angulation of adjacent teeth, measured from CBCT cross-sections using digital angle-measurement tools (Sbricoli et al., 2022; Pippi et al., 2021); patient satisfaction assessed across esthetics, function, comfort, and overall experience; and overall satisfaction scored on a 10-cm visual analog scale (VAS), with 0 representing complete dissatisfaction and 10 representing complete satisfaction (Gönenç and Terzioğlu, 2020; Gonç et al., 2022; Pradyachaipimol et al., 2023; Becerra-Bolaños et al., 2023; Wang et al., 2021). Periodontal and hygiene parameters were also assessed, including the modified plaque index (mPLI) using the Turesky modification of the Quigley-Hein index, the sulcus bleeding index (SBI) according to Saxer and Mühlemann, and probing depth (PD) recorded to the nearest 0.5 mm at four sites around the edentulous area and neighboring teeth. For each index, mean scores per tooth or site were calculated (Mezio et al., 2024).

### 2.6 Statistical analysis

Data were analyzed using IBM SPSS Statistics version 29.0. Normality of continuous variables was tested using the Shapiro-Wilk test. Normally distributed variables were expressed as mean ± standard deviation (SD), and comparisons between groups were performed using independent-sample t-tests, while within-group comparisons at two time points were analyzed using paired t-tests. Non-normally distributed variables were expressed as median (interquartile range) [M (P25, P75)], with group comparisons performed using the Mann–Whitney U test. Categorical variables were expressed as frequencies and percentages [n (%)], with comparisons between groups made using the chi-square test or Fisher’s exact test where appropriate. A p-value <0.05 was considered statistically significant.

## 3 Results

### 3.1 Baseline characteristics

A total of 40 eligible patients were included, with 20 cases in the implant-first cohort and 20 in the orthodontics-first cohort. All participants completed the entire treatment protocol. As shown in Table 1, the mean age was 33.15 ± 6.86 years, with an equal distribution of males (50.0%) and females (50.0%). There were no statistically significant differences between the two cohorts with respect to age, sex, or missing tooth location (*P* > 0.05), indicating that the baseline characteristics were comparable.

**TABLE 1:** Comparison of baseline characteristics.

Variable	Implant-first cohort (n = 20)	Orthodontics-first cohort (n = 20)	t/χ^2^	*P*
Age (years)	32.95 ± 6.71	33.35 ± 7.18	0.182	0.856
Gender			0.000	>0.999
Male	10 (50.0)	10 (50.0)		
Female	10 (50.0)	10 (50.0)		

### 3.2 Tooth position and alveolar bone changes

Both cohorts achieved significant correction of adjacent tooth inclination and sufficient space gain relative to baseline (*P* < 0.05 within cohorts). Between-cohort comparison showed no significant differences in post-treatment inclination correction or final space width (*P* > 0.05), indicating comparable effectiveness in spatial alignment. In contrast, alveolar crest resorption was significantly lower in the implant-first cohort than in the orthodontics-first cohort (0.36 [0.06-0.85] vs. 1.25 [0.54-2.24] mm, *P* = 0.011), plausibly associated with prolonged orthodontic loading in the latter (Table 2). In addition, Table 3 confirmed that before treatment, there were no significant differences in inclination, space width, or alveolar ridge height between the cohorts (*P* > 0.05), supporting that both approaches were effective in uprighting adjacent teeth and achieving the required implant space.

**TABLE 2:** Comparison of preoperative and postoperative indicators between implant-first and orthodontics-first cohorts.

Group	Inclination of teeth adjacent to edentulous space (°)		Width of edentulous space (mm)		Alveolar ridge height (mm)	
	Preoperative	Postoperative	Preoperative	Postoperative	Preoperative	Postoperative
Implant-first cohort (n = 20)	31.05 ± 13.57	8.94 ± 6.50^a^	6.64 ± 4.50	9.03 ± 3.97^a^	13.58 ± 2.76	13.89 ± 3.18
Med [min, max]	26.90 [10.00, 53.00]	6.15 [2.00, 22.80]	5.93 [2.57, 24.03]	7.90 [6.10, 24.64]	13.35 [7.50, 19.30]	13.88 [6.62, 19.39]
Orthodontics-first cohort (n = 20)	30.20 ± 11.01	12.47 ± 7.82^b^	7.27 ± 4.55	8.84 ± 2.41^b^	14.76 ± 2.49	16.34 ± 2.57^b^
Med [min, max]	27.00 [14.10, 49.40]	12.50 [3.00, 29.90]	6.65 [0.00, 20.79]	8.54 [4.19, 15.06]	15.23 [8.78, 18.40]	16.67 [10.20, 19.61]
T	0.218	1.552	0.443	0.188	1.415	2.679
*P*	0.829	0.129	0.660	0.852	0.165	0.011*

*P < 0.05.

^a^ Compared with preoperative values in the implant-first cohort, *P* < 0.05.

^b^ Compared with preoperative values in the orthodontics-first cohort, *P* < 0.05.

**TABLE 3:** Comparison of preoperative-postoperative changes in inclination and width.

Variable	Implant-first cohort (n = 20)	Orthodontics-first cohort (n = 20)	z/t/χ^2^	*P*
Preoperative-postoperative change in inclination (°)	17.00 [13.82, 31.55]	17.35 [10.20, 23.48]	1.096	0.273
Med [min, max]	17.00 [8.00, 41.00]	17.35 [8.20, 34.00]		
Preoperative-postoperative change in width (mm)	2.40 [0.85, 3.80]	1.29 [0.20, 3.85]	1.258	0.208
Med [min, max]	2.40 [-0.02, 6.03]	1.29 [-5.73, 5.38]		
Change in alveolar bone height (mm)	0.36 [0.06, 0.85]	1.25 [0.54, 2.24]	2.516	0.012*
Med [min, max]	0.36 [-0.30, 3.65]	1.25 [-0.22, 5.15]		

**P < 0.01, *P < 0.05.

### 3.3 Treatment efficiency and economic outcomes

The implant-first cohort demonstrated a substantial advantage in treatment efficiency, cost-effectiveness, patient satisfaction, and number of visits (Table 4). Specifically, the time required for localized tooth movement was significantly shorter in the implant-first cohort (5.00 ± 1.25 months) than the time required for space expansion in the orthodontics-first cohort (11.78 ± 2.35 months, *P* < 0.001). The overall treatment cost was significantly lower in the implant-first cohort (¥3,000.00 ± 0.00) compared with the orthodontics-first cohort (¥6,100.00 ± 680.56, *P* < 0.05), largely due to reduced consumption of additional orthodontic materials and fewer follow-up visits. Patient-reported satisfaction, as measured by VAS, was markedly higher in the implant-first cohort (8.05 ± 1.32) compared with the orthodontics-first cohort (6.10 ± 1.68, *P* < 0.001), with most patients highlighting shorter treatment duration, fewer visits, and less discomfort. Similarly, the number of follow-up visits was significantly lower in the implant-first cohort (8.20 ± 1.32) than in the orthodontics-first cohort (15.10 ± 2.49, *P* < 0.001), underscoring the efficiency and patient-centered benefit of the implant-first approach.

**TABLE 4:** Comparison of clinical indicators between cohorts.

Variable	Implant-first cohort (n = 20)	Orthodontics-first cohort (n = 20)	z/t/χ^2^	*P*
Duration of local orthodontic treatment (months)	5.00 ± 1.25	11.78 ± 2.35	11.378	<0.001**
Med [min, max]	5.00 [3.00, 7.00]	12.00 [7.00, 16.00]		
Cost (currency unit)	3,000.00 ± 0.00	6,100.00 ± 680.56	20.371	<0.001**
Med [min, max]	3,000.00 [3,000.00, 3000.00]	6,000.00 [5,500.00, 7500.00]		
Patient satisfaction score	8.05 ± 1.32	6.10 ± 1.68	4.081	<0.001**
Med [min, max]	8.00 [6.00, 10.00]	6.00 [3.00, 9.00]		
Number of follow-up visits	8.20 ± 1.32	15.10 ± 2.49	10.946	<0.001**
Med [min, max]	8.00 [6.00, 10.00]	15.50 [9.00, 19.00]		

**P < 0.01, *P < 0.05.

### 3.4 Patient-reported outcomes and periodontal conditions

Oral hygiene and periodontal health outcomes also favored the implant-first cohort (Table 5). The mean modified Plaque Index (mPLI) was significantly lower in the implant-first cohort (1.20 ± 0.36) compared with the orthodontics-first cohort (3.05 ± 0.50, *P* < 0.01). Similarly, the Sulcus Bleeding Index (SBI) was lower in the implant-first cohort (0.96 ± 0.46) than in the orthodontics-first cohort (1.89 ± 0.39, *P* < 0.001), reflecting reduced gingival inflammation. Probing depth (PD) around implants and adjacent teeth was also significantly shallower in the implant-first cohort (2.09 ± 0.37 mm) than in the orthodontics-first cohort (2.67 ± 0.33 mm, *P* < 0.01), suggesting that shallower periodontal pockets were more conducive to self-maintenance and oral hygiene.

**TABLE 5:** Comparison of periodontal health indicators.

Variable	Implant-first cohort (n = 20)	Orthodontics-first cohort (n = 20)	z/t/χ^2^	*P*
Modified plaque index (mPLI), mean	1.20 ± 0.36	3.05 ± 0.50	13.487	<0.001**
Med [min, max]	1.25 [0.50, 2.00]	3.00 [2.00, 3.75]		
Sulcus bleeding index (SBI), mean	0.96 ± 0.46	1.89 ± 0.39	6.831	<0.001**
Med [min, max]	0.88 [0.25, 1.75]	2.00 [1.25, 2.50]		
Probing depth (PD), mean (mm)	2.09 ± 0.37	2.67 ± 0.33	5.371	<0.001**
Med [min, max]	2.25 [1.50, 2.50]	2.62 [2.25, 3.50]		

**P < 0.01, *P < 0.05.

## 4 Discussion

In this retrospective cohort analysis, two commonly adopted sequencing strategies for managing localized malalignment in partially edentulous patients, an implant-first anchorage pathway and a conventional orthodontics-first pathway, were compared across biomechanical, periodontal, efficiency-related, and patient-reported endpoints. The findings consistently favored the implant-first approach in terms of treatment efficiency, periodontal stability, patient experience, and alveolar preservation, while achieving comparable quality of tooth positional correction to that obtained with orthodontics-first sequencing.

In terms of treatment efficiency, the implant-first strategy demonstrated a clear advantage. The rigid anchorage provided by implants enabled direct and more effective transmission of orthodontic forces to the target teeth, thereby minimizing the limitations associated with conventional anchorage methods such as adjacent teeth, palatal bars, or Nance arches (Mullen, 2023). These traditional approaches often suffer from elastic deformation or anchorage loss, which can reduce efficiency and prolong the treatment course. In contrast, implant-based anchorage reduced the need for complex biomechanical designs and facilitated faster space management, resulting in a shorter overall treatment duration and fewer follow-up visits. Conversely, the orthodontics-first group was more dependent on natural teeth or auxiliary devices for anchorage, which not only increased the risk of anchorage loss but also necessitated longer treatment time and posed greater challenges in controlling adjacent tooth position (Kim et al., 2019).

Periodontal and soft tissue health outcomes also highlighted the potential benefits of the implant-first approach. Because implants served as independent anchorage units, reliance on adjacent teeth was minimized, and fewer auxiliary appliances were required. This translated into lower plaque accumulation, shallower probing depths, and reduced gingival inflammation in the experimental group, as confirmed by significant differences in periodontal indices at the end of treatment (P < 0.01). These findings are clinically important because they underscore that minimizing orthodontic hardware and limiting stress on adjacent teeth can promote better periodontal stability and long-term oral health. Nonetheless, it should be emphasized that regardless of the treatment modality, strict adherence to oral hygiene instructions and regular periodontal maintenance remain essential for sustaining periodontal and peri-implant health over time (Tai et al., 2021; Amerio et al., 2020).

The selection of an optimal treatment strategy must also be guided by patient-specific indications and anatomical considerations. The implant-first approach is particularly suitable for patients with localized edentulism and adjacent teeth presenting mild-to-moderate malpositions, such as mesial inclination, rotation, or extrusion, provided that sufficient bone volume is available and anatomical conditions allow for early implant placement. This strategy integrates orthodontic and restorative phases into a streamlined workflow, ensuring efficient space management, superior anchorage control, and restoration-oriented implant positioning. However, it requires clinicians with advanced surgical and orthodontic expertise who can accurately predict post-orthodontic space distribution and implant site availability, underscoring the importance of interdisciplinary collaboration. In contrast, the orthodontics-first approach remains indispensable in more complex clinical scenarios (Bahamid et al., 2022). Specifically, it is preferable when severe alveolar bone deficiency necessitates prior augmentation, when extensive malocclusion requires large-scale space opening beyond the capacity of localized implant anchorage, or when acute infection or other contraindications preclude immediate implant placement. Under these circumstances, orthodontics-first remains a reliable and effective option, ensuring both treatment safety and long-term stability (Wilhelmy et al., 2022).

Overall, this study highlights that the implant-first approach offers superior efficiency, reduced treatment burden, and enhanced periodontal outcomes, while the orthodontics-first strategy retains its value in specific complex cases. These findings contribute to evidence-based decision-making and reinforce the need for individualized treatment planning, where the choice of strategy is tailored to patient anatomy, clinical complexity, and long-term restorative objectives.

## 5 Conclusion

The findings of this study clearly demonstrate that for patients with localized dentition defects (1-2 teeth) and mild-to-moderate adjacent dentition malformations, the “implant-first” strategy (primary implant placement followed by using the implant as absolute anchorage for localized orthodontic correction after osseointegration) is a more efficient, cost-effective, safe, and esthetically superior comprehensive treatment compared to the conventional “orthodontics-first then implantation” approach. Thus, under strict adherence to indications and standardized clinical procedures, this “implant-first as anchorage” strategy merits clinical promotion to provide patients with more efficient, economical, esthetic, and reliable treatment options, while future studies should further explore its expanded indications, long-term stability, and optimized application of digital technologies (e.g., surgical guides, dynamic navigation) in this combined treatment model.

## 6 Limitations

Several limitations should be acknowledged when interpreting these findings. First, the retrospective design is inherently subject to selection bias and unmeasured confounding, even though baseline comparability was achieved. Second, all treatments were performed at a single institution by experienced specialists, which enhances internal consistency but may limit generalizability to broader practice settings. Third, follow-up was restricted to the completion of prosthetic delivery; therefore, the long-term stability of periodontal and skeletal outcomes under functional loading could not be assessed. Finally, biological parameters such as inflammatory mediators or bone turnover markers were not collected, restricting mechanistic inference.

## Data Availability

The raw data supporting the conclusions of this article will be made available by the authors, without undue reservation.
